# The Utility of an Online Convenience Panel for Reaching Rare and Dispersed Populations

**DOI:** 10.1371/journal.pone.0144011

**Published:** 2015-12-07

**Authors:** Randall Sell, Shoshana Goldberg, Kerith Conron

**Affiliations:** 1 Department of Community Health and Prevention, School of Public Health, Drexel University, Philadelphia, Pennsylvania, United States of America; 2 University of North Carolina, Chapel Hill, North Carolina, United States of America; 3 Center for Population Research in LGBT Health, Fenway Health, Boston, Massachusetts, United States of America; University of Westminster, UNITED KINGDOM

## Abstract

Gaps in data collection systems, as well as challenges associated with gathering data from rare and dispersed populations, render current health surveillance systems inadequate to identify and monitor efforts to reduce health disparities. Using sexual and gender minorities we investigated the utility of using a large nonprobability online panel to conduct rapid population assessments of such populations using brief surveys. Surveys of the Google Android Panel (four assessing sexual orientation and one assessing gender identity and sex assigned at birth) were conducted resulting in invitation of 53,739 application users (37,505 of whom viewed the invitation) to generate a total of 34,759 who completed screening questions indicating their sexual orientation, or gender identity and sex at birth. Where possible we make comparisons to similar data from two population-based surveys (NHIS and NESARC). We found that 99.4% to 100.0% of respondents across our Google Android panel samples completed the screening questions and 97.8% to 99.2% of those that consented to participate in our surveys indicated they were “OK” with the content of surveys that assessed sexual orientation and sex/gender. In our Google Android panel samples there was a higher percentage of sexual minority respondents than in either NHIS or NESARC with 7.4% of men and 12.4% of women reporting gay, lesbian or bisexual identities. The proportion sexual minority was 2.8 to 5.6 times higher in the Google Android panel samples than was found in the 2012 NHIS sample, for men and women, respectively. The percentage of “transgender” identified individuals in the Google sample was 0.7%, which is similar to 0.5% transgender identified through the Massachusetts BRFSS, and using a transgender status item we found that 2.0% of the overall sample fit could be classified as transgender. The Google samples sometimes more closely approximated national averages for ethnicity and race than NHIS.

## Introduction

Responsibility to monitor, protect, and promote the public health is embedded in the United States of America Constitution and is reflected in Healthy People 2020 goals–to “achieve health equity, eliminate disparities, and improve the health of all groups.”[1, 2] Indeed, Healthy People 2020 expresses a commitment to assess “health disparities in the U.S. population by tracking rates of illness, death, chronic conditions, behaviors, and other types of outcomes in relation to demographic factors.”[3] Despite this well-intentioned commitment, gaps in data collection systems, as well as challenges associated with gathering data from rare and dispersed populations, render current health surveillance systems inadequate to identify and monitor efforts to reduce health disparities–particularly those that impact sexual and gender minorities.[4–7]

For example, the National Health Interview Survey (NHIS), considered the “gold standard for U.S. health survey data,” started collecting sexual orientation data in 2012, and, even with a relatively large overall sample size (N = 33,561 asked and answering the sexual orientation question), the first release findings included a comparatively small number of lesbian, gay and bisexual people (n = 571 gay or lesbian, n = 233 bisexual male or female) limiting statistical power to study rare outcomes and to examine heterogeneity across age, race-ethnicity, or geography.

Further, even after aggregating three years (for which gender identity questions were asked) and eight years (for which sexual orientation questions were asked) of Massachusetts Behavioral Risk Factor Surveillance System (BRFSS) state data to achieve samples of more than 30,000 and 60,000 respondents, respectively, only 131 transgender and 2,271 sexual minority respondents were identified due to base rates of 0.5% and 3.0%, respectively. While 2,271 respondents was sufficient to assess and detect effect modification by sexual orientation identity and sex, the sample could not support further exploration by other key characteristics, including ethnicity, race or age. Moreover, the number of health topics that can be reported for sexual and gender minorities who complete BRFSS surveys (as is the case with other surveys that collect data to identify sexual and gender minorities) are limited by randomized survey splits within the survey and variability in items included in surveys over time.

Moreover, the current surveillance system in the Unites States is beleaguered by rising costs associated with declining response rates, and is too sluggish to respond to rapid changes to population health and health determinants.[8] For instance, NHIS releases data on a yearly basis, often up to two years post-collection. Timely health information on populations is needed to achieve the most fundamentals goals of public health including improving quality of life and extending lives. However, representative population data is frequently difficult to collect rapidly and data from rare populations is particularly difficult to collect at any speed. Thus, in order to characterize the health of sexual and gender minorities, other sampling methods must be explored [9–11].

Using sexual and gender minorities (here operationalized as people self-identified as lesbian, gay, bisexual, transgender, genderqueer or gender non-conforming, or for whom current gender and sex at birth differ) we investigated the utility of using a large nonprobability online panel to conduct rapid population assessments and for conducting surveys of rare and dispersed populations. Researchers have proposed that a rare population is one that comprises less than 10% of the overall population, and a dispersed population is one that is intermixed with the general population.[12] Rare and dispersed populations, such as sexual and gender minorities, generally cannot be sampled efficiently using standard sampling techniques. Online panels have been growing rapidly in use and have been receiving considerable attention among survey researchers for surveying rare and dispersed populations. [13, 14] Not only are these types of surveys frequently used, they are used to conduct well designed research that has appeared in leading journals.[15–18] While these panels certainly have significant limitations, which we begin to explore here, they also have appropriate uses which we also examine.[19, 20] The increased use of panel vendors for online survey research makes it essential to better understand their present utility. It is also important to critically examine these methods of data collection because, as they evolve in sophistication, and as people begin to interact in different ways with communication technologies including telephones and the internet, these panels may be the precursor to more perfect survey platforms.[8] Survey methods need to adapt to changes in society and exploit new technologies when they prove valuable to the field.[21, 22]

We believe that online panels may one day be used to collect data: 1) much more rapidly and frequently than more traditional methods of health surveillance allow and on very large samples, 2) from rare and dispersed populations, 3) on a broad set of topics (not limited to topics covered in surveys like the NHIS), 4) that, using appropriate sampling strategies and modeling, is generalizable (e.g. those connected to the internet, or even possibly to general population samples as panels grow in size), 5) at demographic segmentations previously too difficult or costly to sample (e.g. by state to allow for investigations of the impact on health of state employment protection laws which vary across states; or by race and ethnicity within sexual and gender minority populations), and 6) longitudinally allowing for the examination of causation.

To further this goal, using Google Consumer Survey’s Android panel of over one million application installations, we examine: 1) the performance of questions to identify sexual and gender minorities, 2) the prevalence of sexual and gender minorities and their socio-demographic characteristics in this panel, and, 3) how these estimates compare to similar estimates derived from NHIS and the National Epidemiologic Survey on Alcohol and Related Conditions (NESARC) which are considered “gold standard” United States government health surveys and are among the few large government surveys which have produced population-based estimates of sexual minorities.[23, 24]

## Methods

Google Consumer Surveys provided access to the Google Android Panel which served as the sampling frame for this study. The Google Android Panel is one of the options for constructing surveys through the Google Consumer Surveys platform. Google allows for sampling of the android panel and the collection of short surveys (10 items or less). Using the Google Android Panel we experimented with the collection of sexual orientation and gender identity data, as well as other demographic data.

Respondents in the Google Android Panel are users of the Google Opinion Rewards application who have Smart phones operated by Google’s Android operating system and receive small payments of up to one dollar per 10-item survey. Users of Google Opinion Rewards tend to represent earlier-adopters and heavier technology users than on average. As a result, the respondent base is more heavily comprised of younger, male users than the general population. However, Google Opinion Rewards aims when constructing samples from the panel to balance the age, gender and geographic distribution of respondents to closely fit to the distribution of the general population. There were 421,992 installations of the Google Opinion Rewards Application in May 2014 and by May 2015 there were 1,083,391 installations, more than doubling in a year. The Android Google Survey response rate is 98%.

For each survey an individual panel member is sent, Google informs the panel member of how data will be used and asks for their consent. The subject is then compensated with Google Play credit that can be spent in the Google Play Store. Our surveys examining sexual orientation data collection were prefaced with the following statement that allowed respondents to “opt out” of the survey by clicking a button saying “No thanks” or “opt into” the survey by clicking a button saying “OK, got it”: *“This survey will ask you questions about topics that may be sensitive in nature*, *including questions related to sexual orientation and health*. *Answers are completely anonymous and will not be used in any way other than this individual research study*. *By clicking yes you also agree that you are 18 years of age or older*. *Responses to this survey will be aggregated and anonymized with other response and shared with the researcher that created and paid for the survey*.*”*

This work was reviewed by the Institutional Review Board (IRB) of the Drexel University Office of Research which determined that this “is not research involving human subjects as defined by DHHS and FDA regulations.” A Letter of Determination was provided by the IRB to the investigators stating this decision.

The questions used to assess sexual orientation, gender identity, and sex were: 1) *Sexual Orientation—Which of the following best represents how you think of yourself*? *Gay or lesbian*, *Straight*, *that is not gay or lesbian*, *Bisexual*, *Something else*, *I don’t know the answer*, *Refused*, 2) *Gender Identity—What is your current gender identity*? *(Select all that apply) Male*, *Female*, *Transgender*, *Genderqueer/Gender non-conforming*, *I am not sure of my gender identity*, *I do not know what this question is asking*, and 3) *Sex—What sex were you assigned at birth*, *on your original birth certificate*? *Male*, *Female*. The gender identity question allows respondents to self-identify as “transgender” or “genderqueer/gender non-conforming.” Transgender is defined by the American psychological Association as an “umbrella term for persons whose gender identity, gender expression or behavior does not conform to that typically associated with the sex to which they were assigned at birth.”[25] In this paper we also use the term “cisgender” to refer to people whose gender identity conforms with their sex assigned at birth.

The sexual orientation question is currently used on NHIS and underwent extensive testing at the National Center for Health Statistics.[26] Using the same question allows us to compare our findings to NHIS results. The gender identity and sex questions are recommended by the leading experts on transgender health research.[27] From the gender identity and sex questions we constructed a “transgender status” variable which labels respondents reporting: 1) a current gender identity of male and being assigned male at birth as “Male (cisgender)”, 2) a current gender identity of female and being assigned female at birth as “Female (cisgender)”, 3) a current gender identity of male, transgender, or Genderqueer/Gender non-conforming and being assigned female at birth as “Male,Trans,GenQ/Female@Birth”, and 4) a current gender identity of female, transgender, or Genderqueer/Gender non-conforming and being assigned male at birth as “Female,Trans,GenQ/Male@Birth.”

This paper reports on the results of five test surveys that were conducted in April and May, 2015. Using the same survey instrument (which first asked sexual orientation followed by ethnicity and race as well as several health indicators which will be reported elsewhere), we asked Google to: 1) survey males (self-identified as male when signing up for the application, and not based on the second question described above) and use the sexual orientation question to screen for 300 straight identified men who then completed the remainder of the survey, 2) survey males to screen for 600 gay or bisexual identified men, 3) survey females to screen for 300 straight identified women, and 4) survey females to screen for 600 lesbian/gay or bisexual identified women. This strategy of conducting four surveys with screening (rather than a single survey without screening) was chosen in order to reduce respondent burden. The Google Consumer Surveys platform allows users to screen for specific types of subjects based on a single question. In order to identify 600 gay or bisexual men for example, we had to consent 8,678 subjects. If we had not screened, the entire survey would have been administered to over 8000 straight identified men which was many more than required for this exploratory project.

For the fifth survey we asked Google to survey 20,000 respondents who were asked to complete a survey that began with the gender identity and sex questions described above followed by ethnicity and race as well as additional health indicators not reported here. In particular, we report findings here related to the ability of using the Google consumer surveys platform to find rare and disbursed populations.

Where possible we make comparisons to similar data from NHIS (collected in 2013) and NESARC (wave 2 collected in 2004–2005). Both surveys are U.S. cross-sectional household surveys whose data collection methods are described elsewhere. [28, 29]

## Results

Survey response rates are presented in Table 1 which shows the number of people invited to participate in each survey via the delivery of an invite to their device through the number of people who indicated they would take the survey, and finally the number of people that responded to the screening question (i.e. the sexual orientation question for the four sexual orientation surveys, and both the current gender identity and sex at birth questions for the transgender survey).

**Table 1:** Survey Response Rates.

*Survey*	*Survey Invite Delivered to Device*	*Indicated Would “Answer Survey” (Viewed Count)*	*Responded to Consent Question*	*Indicated Would Take Survey*	*Responded to Screening Question(s)*
	n	n (% previous col)	n (% previous col)	n (% previous col)	n (% previous col)
*Straight Male*	456	385 (84.4)	349 (90.6)	346 (99.1)	345 (99.7)
*Gay/Bisexual Male*	11,451	9,173 (80.1)	8,678 (94.6)	8,606 (99.2)	8,564 (99.5)
*Straight Women*	775	450 (58.1)	405 (90.0)	395 (97.5)	395 (100.0)
*Gay/Bisexual Women*	9,469	5,525 (58.3)	5,044 (91.3)	4,976 (98.7)	4,962 (99.7)
*Transgender*	31,588	21,972 (69.6)	21,093 (96.0)	20,620 (97.8)	20,493 (99.4)
*TOTAL*	53,739	37,505 (69.8)	35,569 (94.8)	34,943 (98.2)	34,759 (99.5)

Using the transgender survey as an example (see the second row from the bottom of Table 1), 31,588 people were invited to take the transgender survey by having an invitation delivered to their device (which did not indicate survey content), of which 21,972 people clicked a button saying they would “answer survey” and were consequently presented with a page indicating the nature of the survey and asking for their consent, of which 21,093 responded (473 opting out by saying “no thanks", and 20,620 saying “OK, got it” indicating they would take the survey). Therefore, 879 (21,972 minus 21,093), or 4.0% potentially saw information about the content of the survey and chose to not take it (473 by saying “no thanks” and 406 not responding at all). And finally, 20,493 people responded to both the current gender identity and sex at birth questions which was 93.3% of the “viewed count.”

The survey of gay/bisexual men had an “opt out” rate (those indicating “no thanks” after being informed of the content of the survey) of 0.8% and the survey of straight men had an opt out rate of 0.9%. Women were slightly more likely to opt out of the surveys with an opt out rate of 1.3% for the lesbian/bisexual survey and 2.5% for the survey of straight women. The survey examining gender identity and sex had an opt out rate of 2.2%. The straight male survey was completed by a target sample of 300 in just 21 hours, and the survey of 20,305 people to assess gender identity and sex was completed in just under 60 hours.

Table 2 combines data from the two Google surveys of men assessing sexual orientation, and from the two Google surveys of women assessing sexual orientation, and compares the distribution of sexual orientations identified in the Google surveys to samples available from NHIS (data collected in 2013) and NESARC (wave 2 collected in 2004–2005).

**Table 2:** Sexual Orientation Distributions for a Google Android Panel Sample, NHIS and NESARC.

*Sexual Orientation*	*Male Google*	*NHIS*	*NESARC*	*Female Google*	*NHIS*	*NESARC*
	n (%)	n (%)	n (%)	n (%)	n (%)	n (%)
*Straight*	7,638 (89.2)	14,495 (96.6)	14,109 (97.6)	4,073 (82.1)	18,051 (97.6)	19,489 (98.0)
*Gay or Lesbian*	309 (3.6)	320 (2.1)	190 (1.3)	145 (2.9)	251 (1.4)	145 (0.7)
*Bisexual*	326 (3.8)	78 (0.5)	81 (0.6)	473 (9.5)	155 (0.8)	161 (0.8)
*Something else*	153 (1.8)	29 (0.2)		172 (3.5)	27 (0.1)	
*I don’t know*	138 (1.6)	76 (0.5)	69 (0.5)	99 (2.0)	79 (0.4)	101 (0.5)
*Total*	8,564 (100.0)	14,998 (100.0)	14,449 (100.0)	4,962 (100.0)	18,563 (100.0)	19,896 (100.0)

Table 3 shows the distribution of gender identity and sex at birth as well as our “transgender status” variable from the Google survey of gender identity and sex. Only subjects completing the entire survey are included in this analysis resulting in 20,305 subjects which is 188 less than the 20,493 subjects reported in Table 1.

**Table 3:** Google Android Panel Reported Gender Identity and Sex at Birth, and Computed Transgender Status.

*Gender Identity*		*Sex at Birth*		*Transgender Status*	
	n (%)		n (%)		n (%)
*Male*	9,966 (49.1)	*Male*	10,208 (50.3)	*Male (cisgender)*	9,815 (48.3)
*Female*	9,931 (48.9)	*Female*	10,097 (49.7)	*Female (cisgender)*	9,733 (47.9)
*Transgender*	149 (0.7)			*Female*,*Trans*,*GenQ/Male@Birth*	187 (0.9)
*Genderqueer*	245 (1.2)			*Male*,*Trans*,*GenQ/Female@Birth*	225 (1.1)
*Other*	382 (1.9)			*Other*	345 (1.8)
*Total*	20,305 (100.0)	*Total*	20,305 (100.0)	*Total*	20,305 (100.0)

Tables 4, 5, 6 and 7 present ethnic and racial distributions respectively from the Google surveys of sexual orientation in comparison with findings from NHIS 2013 data.

**Table 4:** Google Android Panel Samples and the NHIS Reported Ethnicity for Men by Sexual Orientation.

*ETHNICITY*	*Straight Google*	*NHIS*	*Gay Google*	*NHIS*	*Bisexual Google*	*NHIS*
	n (%)	n (%)	n (%)	n (%)	n (%)	n (%)
*Hispanic*	33 (11.3)	1,156 (16.6)	60 (20.1)	31 (19.7)	59 (20.1)	1 (2.9)
*Not Hispanic*	260 (88.7)	5,795 (83.4)	238 (79.9)	126 (80.3)	234 (79.9)	33 (97.1)
*Total*	293 (100.0)	6,951 (100.0)	298 (100.0)	157 (100.0)	293 (100.0)	34 (100.0)

**Table 5:** Google Android Panel Samples and the NHIS Reported Ethnicity for Women by Sexual Orientation.

*ETHNICITY*	*Straight Google*	*NHIS*	*Gay Google*	*NHIS*	*Bisexual Google*	*NHIS*
	n (%)	n (%)	n (%)	n (%)	n (%)	n (%)
*Hispanic*	31 (10.4)	1,531 (17.3)	27 (19.3)	17 (13.7)	82 (18.4)	11 (16.4)
*Not Hispanic*	267 (89.6)	7,313 (82.7)	113 (80.7)	107 (86.3)	364 (81.6)	56 (83.6)
*Total*	298 (100.0)	8,844 (100.0)	140 (100.0)	124 (100.0)	446 (100.0)	67 (100.0)

**Table 6:** Google Android Panel Samples and the NHIS Reported Race for Men by Sexual Orientation.

*RACE*	*Straight Google*	*NHIS*	*Gay Google*	*NHIS*	*Bisexual Google*	*NHIS*
	n (%)	n (%)	n (%)	n (%)	n (%)	n (%)
*White Only*	196 (66.9)	5,325 (76.6)	206 (69.1)	129 (82.2)	185 (63.1)	23 (67.7)
*Black/African American Only*	18 (6.1)	967 (13.9)	19 (6.4)	13 (8.3)	11 (3.8)	6 (17.7)
*Asian Only*	21 (7.2)	451 (6.5)	16 (5.4)	8 (5.1)	17 (5.8)	5 (14.7)
*Some other race Only*	26 (8.9)	84 (1.2)	20 (6.7)	1 (0.6)	29 (9.9)	0 (0.0)
*Multi-Race*	32 (10.9)	124 (1.8)	37 (12.4)	6 (3.8)	51 (17.4)	0 (0.0)
*Total*	293 (100.0)	6,951 (100.0)	298 (100.0)	157 (100.0)	293 (100.0)	34 (100.0)

**Table 7:** Google Android Panel Samples and the NHIS Reported Race for Women by Sexual Orientation.

*RACE*	*Straight Google*	*NHIS*	*Gay Google*	*NHIS*	*Bisexual Google*	*NHIS*
	n (%)	n (%)	n (%)	n (%)	n (%)	n (%)
*White Only*	220 (73.8)	6,547 (74.0)	88 (62.7)	90 (72.6)	275 (61.7)	51 (76.1)
*Black/African American Only*	14 (4.7)	1,468 (16.6)	14 (10.0)	22 (17.7)	33 (7.4)	8 (11.9)
*Asian Only*	16 (5.4)	542 (6.1)	5 (3.6)	3 (2.4)	17 (3.8)	0 (0.0)
*Some other race Only*	21 (7.0)	117 (1.3)	12 (8.6)	1 (0.8)	36 (8.2)	2 (3.0)
*Multi-Race*	27 (9.1)	170 (1.9)	21 (15.0)	8 (6.5)	85 (19.0)	6 (9.0)
*Total*	298 (100.0)	8,844 (100.0)	140 (100.0)	124 (100.0)	446 (100.0)	67 (100.0)

Tables 8 and 9 present ethnic and racial distributions respectively from the Google samples for the transgender status variable. Comparisons for Tables 8 and 9 to other surveys (such as NHIS) are not provided here because such data does not exist.

**Table 8:** Ethnicity and Transgender Status in a Google Android Panel Sample.

*RACE*	*Male (cisgender)*	*Female (cisgender)*	Female,Trans,GenQ/ Male@Birth	Male,Trans,GenQ/ Female@Birth
	n (%)	n (%)	n (%)	n (%)
*Hispanic*	1,213 (12.4)	1,186 (12.2)	51 (22.7)	56 (30.0)
*Not Hispanic*	8,602 (87.6)	8,547 (87.8)	174 (77.3)	131 (70.0)
*Total*	9,815 (100.0)	9,733 (100.0)	225 (100.0)	187 (100.0)

**Table 9:** Race and Transgender Status in a Google Android Panel Sample.

*RACE*	*Male (cisgender)*	*Female (cisgender)*	Female,Trans,GenQ/ Male@Birth	Male,Trans,GenQ/ Female@Birth
	n (%)	n (%)	n (%)	n (%)
*White Only*	6,493 (68.2)	6,618 (70.0)	113 (52.6)	86 (48.0)
*Black/African American Only*	468 (4.9)	707 (7.5)	12 (5.6)	10 (5.6)
*Asian Only*	976 (10.3)	633 (6.7)	15 (7.0)	8 (4.5)
*Some other race Only*	488 (5.1)	433 (4.6)	18 (8.4)	24 (13.4)
*Multi-Race*	1,094 (11.5)	1,069 (11.3)	57 (26.5)	51 (28.5)
*Total*	9,519 (100.0)	9,460 (100.0)	215 (100.0)	179 (100.0)

## Discussion

Large online panels such as the Google Android panel present an extraordinary opportunity to investigate the health of rare and dispersed populations such as sexual and gender minorities. Simply asking sexual orientation and gender identity is a relatively recent occurrence in survey research and for that matter, in research in general. Even today many survey administrators are hesitant to add such questions to their surveys because they believe the questions would “offend” some respondents resulting in survey non completion or requests by participants to be removed from panels. This was one of the primary reasons NHIS waited almost 25 years to collect sexual orientation data after first being asked to do so. However, numerous surveys have now collected this data demonstrating that the overwhelming majority of people in the context of a research study are willing to respond to a sexual orientation question.[10] Unfortunately, less research has been done looking at the impact of gender identity and sex at birth questions on survey completion and nonresponse, however, there is little reason to believe respondents would have any more trouble with these questions than sexual orientation questions.[27]

Few people in our surveys seemed to have a problem with the content of the surveys. We found, of those that indicated they would take the survey, that 99.4% to 100.0% of respondents across our surveys completed the screening questions and 97.8% to 99.2% of those that responded to the consent question indicated they were “OK” with the content of the survey and wanted to take it.

In our Google Android panel samples there was a higher percentage of non “straight” respondents than in either NHIS or NESARC with 7.4% of men and 12.4% of women reporting gay, lesbian or bisexual identities. This was 2.8 times higher than found in the 2012 NHIS sample for men and 5.6 times higher for women. This in part may be explained by the younger and more educated sample from Google, but NHIS may also be achieving lower non-straight identity rates because it is a government survey and people may be hesitant to disclose their sexual orientation to the United States federal government even when promised confidentiality. NHIS may also be impacted by the survey format that uses face-to-face questioning, while the Google android panel uses Android devices as interfaces for collecting self-report data. In any case, many less people needed to be surveyed in the Google Android panel samples to identify the same number of gay, lesbian, and bisexual people as NHIS or NESARC.

The percentage of “transgender” identified individuals in the Google sample was 0.7% which was slightly higher than found in Massachusetts BRFSS data, and using our transgender status variable we found that 0.9% of the sample reported current female, transgender or gender queer identities and being assigned male at birth, and 1.1% reported current male, transgender or gender queer identities and being assigned male at birth. Using this measure of transgender status results in 2.0% of the overall sample fitting within a label of transgender (see Table 3).

As of July 1, 2013, the United States Census Bureau reports that 17.1% of the United States population identifies as Hispanic or Latino, while racially, 77.7% identify as white alone, 13.2% identify as Black or African American alone, and 5.3% identify as Asian alone.[30] Table 4 shows that our Google male samples were a little less Hispanic identified than the general population while the gay and bisexual Google samples were a little more likely to identify as Hispanic. The NHIS straight and gay male samples more closely resembled the prevalence of the general population identifying as Hispanic while the NHIS bisexual male sample was dramatically lower (2.9%). For women (see Table 5) there was similar variability in the Hispanic identity across the samples with the Google samples sometimes being closer to the Census estimates than NHIS estimates and sometimes further away. This variability for men and women is most likely due to the small sample sizes in both the Google and NHIS surveys but also to the survey sampling frames which have unique biases. While we would expect the NHIS samples to more closely mirror Census estimates that was not always the case.

Tables 6 and 7 show that the samples identified through Google were generally less White and less Black/African-American than NHIS and less so than the prevalence of White or Black/African American people in the United States. Only the prevalence of Black/African-American lesbian women exceed the national average. The Google samples more closely approximated the national average for the prevalence of Asian men and women for the straight, gay, and bisexual categories.

In a transgender survey we find that both cisgender male and female individuals were slightly less likely to be Hispanic while the transgender individuals, using our transgender status categories, were much more likely to report Hispanic identity. Similarly, transgender individuals (see Table 9) were much less likely to be white identified than cisgender individuals. We do not provide a comparison In Table 9 to the NHIS or any other federal survey because few if any good comparisons are available for national samples of transgender people, but the Google samples of gender minorities are less White than the general United States population.

While large online convenience panel samples like the one examined here have a number of limitations, their ability to collect data rapidly on a large number of rare and dispersed people that are similar to the general population has significant advantages that need to be explored. Here we demonstrated the ability to rapidly and cheaply collect data on sexual orientation, gender identity, and sex assigned at birth, and to produce samples from which additional data can be collected to assess important topics which have previously been unexplored in these populations. Further, we believe that with the proper pre-and post-stratification to correct for the convenience nature of the sampling strategy, these samples can be weighted to provide accurate representations of the prevalence and characteristics of rare and dispersed populations.

## Supporting Information

S1 Data This file contains the data analyzed for this paper. (XLS)
